# Human-to-Dog Transmission of Methicillin-Resistant *Staphylococcus aureus*

**DOI:** 10.3201/eid1012.040387

**Published:** 2004-12

**Authors:** Engeline van Duijkeren, Maurice J.H.M. Wolfhagen, Adrienne T.A. Box, Max E.O.C. Heck, Wim J.B. Wannet, Ad C. Fluit

**Affiliations:** * Faculty of Veterinary Medicine, Utrecht, the Netherlands;; † Isala Clinics, Zwolle, the Netherlands;; ‡ University Medical Center Utrecht, Utrecht; the Netherlands;; § National Institute of Public Health and the Environment, Bilthoven, the Netherlands

**Keywords:** methicillin resistant Staphylococcus aureus, dog, human, transmission, dispatch

## Abstract

Methicillin-resistant *Staphylococcus aureus* (MRSA) was cultured from the nose of a healthy dog whose owner was colonized with MRSA while she worked in a Dutch nursing home. Pulsed-field gel electrophoresis and typing of the staphylococcal chromosome cassette *mec* (SCC*mec*) region showed that both MRSA strains were identical.

## The Case

In 2000, a methicillin-resistant *Staphylococcal aureus* (MRSA) strain was isolated from a patient admitted to a multisite 1,100-bed tertiary-care teaching hospital in the Netherlands. This strain recurred during several outbreaks and spread to a 190-bed nursing home, which is part of the hospital. During a large outbreak in 2003 in the nursing home, 48 patients and 15 nurses were identified as carriers of MRSA, either in their nares, throat, perineum, or a combination of these sites. All MRSA isolates from the outbreaks were sent to the National Institute of Public Health and the Environment (RIVM) for identification and genotyping by pulsed-field gel electrophoresis (PFGE). Most MRSA had the same PFGE pattern, RIVM cluster 35.

In 2003, a 31-year-old female nurse who had psoriasis was identified as an MRSA carrier during the above-mentioned MRSA outbreak. The nurse was treated to eliminate MRSA carriage by applying mupirocin ointment in her nares and washing with a chlorhexidine in ethanol solution for 7 days. Initially she became MRSA-negative but later converted to a carrier state again: samples from her nose, throat, perineum, and skin lesions were taken, and MRSA could be isolated from all sites. She was treated for her psoriasis with topical application of triamcinolonacetonide/tetracycline to minimize the skin lesions and thereafter with oral doxycycline and rifampin to eliminate MRSA. However, after some weeks, she became colonized again at all previously mentioned sites. Screening her home environment showed that her 1-year-old daughter, who also had psoriasis, was colonized in the nose, throat, and skin lesions. The nares, but not the perineum, of their healthy pet dog were also colonized. The dog had not been treated with antimicrobial drugs in the past. Samples from nose, throat, and perineum from the patient's husband were MRSA negative, as were samples from nose, throat, perineum, and skin lesions of the baby's grandmother, who also had psoriasis and took care of the baby when the mother worked.

The staphylococci isolated from the nurse, her child, and the dog were all identified as *S. aureus* by conventional methods, Vitek 2 (BioMérieux, Marcy-l'Etoile, France) and Martineau polymerase chain reaction (PCR), which targets the *tuf* gene (*1*). Susceptibility testing was performed by using the Vitek 2, according to the manufacturer's instructions. The isolates were resistant to penicillin G, ofloxacin, trimethoprim-sulfamethoxazole, and fusidic acid and tested susceptible to aminoglycosides, tetracyclines, erythromycin, clindamycin, vancomycin, rifampin, and oxacillin. Disk diffusion testing demonstrated susceptibility to mupirocin. Because the *mecA*-positive MRSA isolates from previous outbreaks in the nursing home were also resistant to ofloxacin, trimethoprim-sulfamethoxazole, and fusidic acid, and tested either oxacillin resistant or oxacillin susceptible by Vitek 2, the oxacillin resistance of our isolates was also tested by several other methods. The oxacillin screening test (Mueller-Hinton agar supplemented with 4% NaCl containing oxacillin at a concentration of 6 μg/mL) showed no growth after 24 h and 48 h of incubation at 35°C. However, the Etest (AB Biodisk, Solna, Sweden) showed that the isolates of the dog and the baby had an oxacillin MIC of 6 mg/L and the isolate from the nurse had an oxacillin MIC of 4 mg/L and could therefore be classified as oxacillin resistant. In addition, a PBP2 slide latex agglutination test (Oxoid, Haarlem, Netherlands) was positive for all three isolates. The presence of the *mecA* gene (*2*) was demonstrated by a positive *mecA* PCR for all three isolates.

Characterization of the staphylococcal chromosome cassette *mec* (SCC*mec*) was performed by typing the *ccrA/B* gene complex, the *mec* complex, and by means of a PCR strategy, which detects structural variants of SCC*mec* covering the entire genetic element (*2**–**6*). Typing the SCC*mec* of the MRSA strains cultured from the dog and its owner showed that these seemed to be identical (*ccrA/B* gene type 4, *mec* complex class B, and no loci A-H were found).

PFGE was carried out as described by Schwarzkopf et al. (*7*). PFGE showed that all three MRSA isolates (from the dog, the child, and the nurse) had indistinguishable patterns and that they belonged to RIVM cluster 35, an epidemic human MRSA cluster. This cluster was the same one cultured previously from patients and contacts in the nursing home during several outbreaks since 2000. On the basis of these data, we assume that the dog became colonized with the same strain as its owner through contact, and that either the dog or the baby or an unknown source within the nursing home reinfected the nurse. We finally treated mother and child for the skin lesions simultaneously as mentioned before. Afterward, the mother and the dog received an oral course of doxycycline and rifampin; the baby was treated with clarithromycin and rifampin. This treatment finally eliminated MRSA from the mother, the child, and the dog. Topical application of antimicrobial drugs in dogs is impractical. Follow-up cultures from the dog and the child were taken for 2 months, the mother was monitored for 9 months, and all cultures remained negative.

## Conclusions

MRSA is an important cause of human nosocomial and community-acquired infections worldwide. In contrast, few cases of MRSA infections in dogs have been reported (*8**–**11*). We report the first known case of human-to-animal transmission of MRSA in the Netherlands. In the United States, the transmission of MRSA between a pet dog and its owners has been reported (*12*): a patient with diabetes had recurrent infections of the leg with a mupirocin-resistant MRSA strain, and his wife had cellulitis. Culture from their dog's nose grew an MRSA isolate with the same antimicrobial-resistance pattern and an identical PFGE pattern as the isolate cultured from wounds and nares of the couple. Further recurrence of the MRSA infection of the couple was only prevented when the dog was no longer an MRSA carrier (*12*). Cefai et al. (*13*) isolated an MRSA strain with an identical phage type from the nose of a male nurse, his wife, and their pet dog. Transmission of MRSA between humans and horses has also been suspected in a veterinary teaching hospital in the United States (*14*).

SCC*mec* is a mobile genetic element that carries the *mecA* gene, which mediates methicillin resistance in staphylococci. To date, four SCC*mec* types have been described (*2**,**3**,**6*). Typing the SCC*mec* of the MRSA in our study showed that they were of an uncommon type, which could not be classified as one of the four SCC*mec* types. Oliveira et al. (*2*) defined SCC*mec* type IV as harboring *ccrA/B* type 4 in combination with characteristic loci, whereas Ito et al. (*3*) defined SCC*mec* type IV as the unique combination of the class B *mec* and type 2 *ccr* gene complex. Our MRSA had *ccrA/B* type 4 and class B *mec* but no loci. Therefore, this MRSA cannot be classified in the current system and may present a new SCC*mec* type. This new type may spread successfully, as has been seen with SCC*mec* types I–IV. During the 1960s, MRSA carrying SCC*mec* type I spread across the world, followed by a second wave of MRSA during the late 1970s that carried SCC*mec* II; strains with SCC*mec* III spread during the 1980s, and strains with SCC*mec* type IV have been isolated worldwide since the beginning of the 1990s. Recently, an isolate with a *ccr*5 type was described (*15*), which indicates that additional SCC*mec* types are present in *S. aureus*. Isolates with new SCC*mec* types may be the frontrunners of new waves of MRSA, posing an unknown health threat.

Automated systems such as Vitek 2 are generally reported to be reliable for testing methicillin or oxacillin susceptibility, but the Vitek 2 did not detect MRSA in our study. Misclassification of *mecA*-positive *S. aureus* as oxacillin susceptible by the Vitek 2 has been reported before by Felten et al. (*16*), especially in strains with relatively low oxacillin MICs. The oxacillin-screening test also did not detect our MRSA strains. This can be explained by their relatively low oxacillin MICs (4 mg/L and 6 mg/L, respectively). The most reliable procedure for detecting MRSA remains the PCR amplification of the *mecA* gene. MRSA in this study was resistant to fluoroquinolones, which is common in MRSA but not in methicillin-susceptible *S. aureus* (*17*). Therefore, fluoroquinolone-resistant *S. aureus* strains should always be suspected of being MRSA and should be tested for the presence of the *mecA* gene by PCR.

In conclusion, dogs and other pets living in close contact with human MRSA carriers can become colonized with MRSA. Failure to detect and treat these colonized pets can result in recurrent MRSA colonization or infection in humans. Therefore, the risk of pets being the source of unexplained carriage or relapse of infection in humans should be recognized. Antimicrobial therapy of healthcare workers and, at the same time, of MRSA carriers and any infected family members or pets, can eliminate recurrent MRSA carriage. Pets should be treated systemically, since topical application is impractical.
